# p63^+^Krt5^+^ basal cells are increased in the squamous metaplastic epithelium of patients with radiation-induced chronic Rhinosinusitis

**DOI:** 10.1186/s13014-020-01656-7

**Published:** 2020-09-25

**Authors:** Hongming Huang, Kai Sen Tan, Suizi Zhou, Tian Yuan, Jing Liu, Hsiao Hui Ong, Qianmin Chen, Junxiao Gao, Minghong Xu, Zhenchao Zhu, Qianhui Qiu, De Yun Wang

**Affiliations:** 1 Department of Otolaryngology, Guangdong Provincial People’s Hospital and Guangdong Academy of Medical Sciences, Guangzhou, China; 2 Department of Otolaryngology, National University of Singapore, 1E Kent Ridge Rd, Singapore, 119228 Singapore; 3 Department of Otolaryngology, Zhujiang Hospital, Southern Medical University, Guangzhou, 510282 China; 4 Department of Otolaryngology, The Third Affiliated Hospital of Sun Yat-Sen University, Guangzhou, China

**Keywords:** Squamous metaplasia, Epithelium, Basal cells, Radiation, Chronic Rhinosinusitis(CRS)

## Abstract

**Background:**

Squamous metaplasia (SM) is an irreversible form of airway epithelial remodeling. Hyperproliferation of basal cells was observed in squamous metaplastic epithelium of chronically inflamed airway. However, the association of such aberrant proliferation of basal cells with SM in the nasal epithelium after radiation damage remains unclear. The aim of this study was to investigate SM and accompanying levels of p63^+^Krt5^+^ (basal cell markers) cells in the nasal epithelium of patients with radiation-induced chronic rhinosinusitis (CRSr) and patients with chronic rhinosinusitis without nasal polyps (CRSsNP) compared to healthy controls.

**Methods:**

We assessed the prevalence of SM and the expression of p63^+^, Krt5^+^, p63^+^Krt5^+^, and Ki67^+^ cells through immunofluorescence(IF) staining of the inferior turbinate (IT) tissues from patients with CRSr (*n* = 36), CRSsNP (*n* = 33) and controls (*n* = 28).

**Results:**

The prevalence of SM and the number of p63^+^Krt5^+^ cells were both significantly increased in patients with CRSr compared to patients with CRSsNP and controls. The number of Ki67^+^ cells were both significantly increased in patients with CRSr and CRSsNP compared to controls, but the ratio of Ki67^+^ cells to p63^+^Krt5^+^ cells was significantly lower in patients with CRSr compared to patients with CRSsNP. In patients with CRSr, an increased number of p63^+^Krt5+ basal cells was observed in SM epithelium compared to non-SM epithelium.

**Conclusion:**

SM is increased in the nasal epithelium of patients with CRSr, in which aberrant levels of p63^+^Krt5^+^ basal cells serves as an important pathologic feature in the squamous metaplastic epithelium.

## Introduction

Nasopharyngeal carcinoma (NPC) is one of the most common cancers in the head and neck region, and the incidence of NPC is remarkably high in Southern China at up to 25 per 100,000 [1]. NPC is highly radiosensitive, and radiotherapy (RT) is the mainstay for its treatment [2]. While RT is effective, chronic rhinosinusitis (CRS) often arise as one of the most common side-effect of the treatment in NPC patients. The incidence of radiation-induced CRS (CRSr) in NPC patients after RT ranged from 43.2 to 73.5% [3, 4]. Abnormal ciliary ultrastructure and mucociliary function were widely found in the nasal epithelium of NPC patients after RT, which is the leading cause of CRSr [5–7]. Unlike conventional CRS, the treatment of CRSr remains a challenge on account of the lack of effective method to restore the structure and function of irradiated nasal epithelium.

Epithelial remodeling is a crucial pathological feature of chronic airway inflammatory diseases, including three main forms: epithelial hyperplasia, goblet cell hyperplasia, and squamous metaplasia (SM) [8, 9]. SM is a severe and irreversible form of epithelial remodeling, characterized by the replacement of normal columnar epithelium by squamous epithelium [10]. SM represents a transition from the normal ciliated pseudostratified columnar epithelium of the respiratory mucosa to a nonkeratinized squamous epithelium [11]. SM are commonly presented in chronic upper airway disease, at about 18% in routine CRS and 44.6% in nasal polyps (NPs) specimens [11, 12]. The SM areas are characterized with absence of normal ciliated cell and goblet cell structure, resulting in dysfunctions in ciliary clearance and secretions in the nasal epithelium [12]. However, so far few studies have focused on the epithelial remodeling in patients with CRSr.

A healthy nasal epithelium is composed of four intrinsic cell types: basal cells, goblet cells, ciliated cells and non-ciliated columnar cells [13]. Basal cells, which have a high proliferative and differentiation capacity, are regarded as the stem/progenitor cells of airway epithelium and play a critical role in epithelial repair [14]. In response to the injury of the airway epithelium, basal cells can proliferate and migrate to the damaged site denuded of differentiated epithelial cells and subsequently differentiate to restore a healthy epithelial cell layer (columnar or goblet cells) [15]. A comprehensive set of markers was established to access the morphology and function of nasal epithelial cells, including p63 (basal cell), Krt5(basal cell), MUC5AC(goblet cell), acetylated alpha-tubulin (ciliated cell) and Ki67 (proliferating cell) in the nasal epithelium [14]. In a recent study, we found an increase of poorly proliferated p63^+^/Ki67^+^ basal cells in squamous metaplastic epithelium from patients with NPs, suggesting that pathologic proliferation of basal cells may play an important role in remodeled epithelium from NPs [16]. On the other hand, the etiology and mechanism of epithelial remodeling in CRSr epithelium after RT remains understudied.

In this study, we sought to investigate the prevalence of epithelial remodeling in nasal epithelium from patients with CRSr and explore the similarities and differences in the aberrant proliferation of basal cells involved in the remodeling process of nasal epithelium after RT. The objective of the study is to assess the difference in treatment needs for CRSr compared to conventional CRS treatments. It is hoped that the results of this study will provide new insights into the molecular mechanism underlying epithelial remodeling in CRSr, which may be helpful for clinical treatment of CRSr.

## Materials and methods

### Patient recruitment and ethics statement

This study was approved by the institutional review boards of the Guangdong Provincial People’s Hospital (China), the Zhujiang Hospital of Southern Medical University (China), and the National Healthcare Group Domain-Specific Review Board of Singapore (Singapore). Healthy controls(*n* = 28) were recruited from subjects undergoing septoplasty due to deviation of nasal septum; while patients with CRS without nasal polyps (CRSsNP) (*n* = 33) and patients with CRSr (*n* = 36) were recruited from subjects undergoing functional endoscopic sinus surgery (FEES) in these participating hospitals. The patients with CRSr in this study were all recruited from NPC patients who underwent a standard-course of intensity-modulated radiotherapy (IMRT). All the NPC patients were restaged according to the 7th edition of the Union International Centre le Cancer /American Joint Committee on Cancer (UICC/AJCC) system [17]. The primary tumor received standard-course radiotherapy to a dose of 66 to 70 Gy in 33 to 35 fractions, 2 Gy per fraction. The neck was treated to a dose of 60 Gy in 30 fractions, 2 Gy per fraction. All fields were treated once daily, 5 times a week. The total radiation dose received at the middle of the IT was approximately 5000GY. The diagnosis of CRSsNP and CRSr was made according to the current European position paper (EPOS) on rhinosinusitis and nasal polyps (EPOS 2012) [18], based on medical history, electronic nasopharyngoscopy and computed tomography (CT) scans, and confirmed by the postoperative histopathological report. NPC patients who had CRS before RT were excluded from the study. Biopsies of the middle of inferior turbinate (IT) mucosa were taken from all the recruited subjects during surgery. The details of the subjects are presented in Table 1.

**Table 1:** Summary of patient characteristics and the methods

	Controls	CRSsNP	CRSr	***p-***value^*^
**Sample size**	**28**	**33**	**36**	**–**
**Age, years[mean ± SD]**	**45.2 ± 12.5**	**48.8 ± 15.2**	**49.7 ± 11.9**	**n.s**
**Male/female**	**22/6**	**20/13**	**27/9**	**n.s**
**smoker/nonsmoker**	**8/20**	**11/22**	**15/21**	**n.s**
**Methodology used**				
Paraffin specimens	**28**	**33**	**36**	**–**
H&E	**28**	**33**	**36**	**–**
IF	**28**	**33**	**36**	**–**
qRT-PCR	**6**	**0**	**20**	
**Epithelial remodeling [No. (%)]**^**a**^				
Epithelial hyperplasia	**0(0)**	**7(21.2)**	**10(27.8)**	
Goblet cell hyperplasia	**0(0)**	**3(9.1)**	**5(13.9)**	
Squamous metaplasia	**0(0)**	**2(6.1)**	**13(36.1)**	

*n.s* not signficant

Values are n or mean ± SD

*H&E* Hematoxylin and eosin, *IF* immunofluorescence

*The level of significance was evaluated using T test or Fisher exact test. *P* < 0.05 was considered statistically significant

^a^Epithelial hyperplasia was defined as epithelium with more than 4 layers of cells. Goblet cell hyperplasia was defined as 2 or more layers of goblet cells in the epithelium. Squamous metaplasia was identified in specimens where the epithelium had lost its pseudostratified columnar epithelial structure with absence of goblet cells and cilia and was replaced by squamous epithelium

### Hematoxylin and eosin(H&E) staining and IF staining

All IT biopsy specimens were fixed in formalin and embedded in paraffin. Paraffin sections were sectioned at 4 μm thickness using Leica microtome (Leica, Wetzlar, Germany). We performed H&E staining to assess the epithelium of all specimens. Subjects without epithelium in specimens were excluded from this study.

Specimens were also assessed by IF staining for ciliated cell marker (acet. α-tubulin), goblet cell marker (MUC5AC), basal cell markers (p63 and Krt5) and cell proliferation marker (Ki67). The details of the primary antibodies for IF staining were described in Table 2. Briefly, the sections were dewaxed, blocked, and then incubated with the primary antibody overnight at 4 °C. Specimens were then incubated with Alexa Fluor 488- or 594-conjugated secondary antibody (Life Technologies, Carlsbad, CA, USA) in the dark at room temperature for 1 h. Coverslips were mounted on the slides using Slow Fade Gold antifade reagent with 4′6-diamidino-2-phenylindole (Life Technologies, Carlsbad, CA). For negative controls, the primary antibodies were substituted with species- and subtype-matched antibodies at the same concentration. The slides were examined with a fluorescent microscopy (Olympus IX51, Tokyo, Japan).

**Table 2:** Primary antibodies for IF staining

Primary antibody	Description	Supplier	Dilution rate
acet.α-tubulin	Rabbit monoclonal [EP1332Y] to alpha Tubulin (ab 52,866)	Abcam,Cambridge, UK	1:1000
MUC5AC	Mouse monoclonal[2-11 M1] to MUC5AC (ab 24,071)	Abcam,Cambridge, UK	1:600
p63	Mouse monoclonal [4A4] to p63 (ab 735)	Abcam,Cambridge, UK	1:100
Krt5	Rabbit monoclonal [EP1601Y] to Krt5 (ab 52,635)	Abcam,Cambridge, UK	1:600
Ki67	Rabbit monoclonal [SP6] to Ki67 (ab 1667)	Abcam,Cambridge, UK	1:400

### Histologic evaluation

#### Evaluation of epithelial remodeling

The assessment of epithelial remodeling was performed by H&E and IF staining. Epithelial hyperplasia was defined as epithelium with more than 4 layers of cells. Goblet cell hyperplasia was defined as 2 or more layers of goblet cells in the epithelium. Squamous metaplasia was identified in specimens where the epithelium had lost its pseudostratified columnar epithelial structure with absence of goblet cells and cilia, and was replaced by squamous epithelium [12]. All Specimens were evaluated by 2 independent examiners following the same protocol, which had achieved consistent results.

#### Evaluation of IF staining for epithelial cell markers

Two researchers independently assessed all cases in a blinded manner to have a standardized histologic evaluation of the IF staining. Three areas from the tissue sections were randomly selected at × 400 magnification with a fluorescence microscope. Expression of epithelial cell markers were quantified with ImageJ software.

Cilia and goblet cell in nasal epithelium were evaluated by assessing the positive staining area of acet. α-tubulin and MUC5AC, respectively. The mean value of three areas was calculated for each paraffin-embedded section.

The p63^+^, Krt5^+^, p63^+^Krt5^+^, and Ki67^+^ cells within the epithelial region were counted in three individual fields, respectively. Expression levels of p63^+^Krt5^+^ and Ki67^+^ cells were calculated as an average of the positive cells. The ratio of Ki67^+^ cells to p63^+^Krt5^+^ cells and p63^+^ cells to Krt5^+^ cells were then calculated. As we observed asynchronous expression of p63^+^ and Krt5^+^ cells in SM, we further classified SM into four patterns based on the ratio of p63^+^ cells to Krt5^+^ cells in nasal epithelium: (1) Pattern A, the ratio of p63^+^ cells to Krt5^+^ cells ≥80%; (2) Pattern B, the ratio of p63^+^ cells to Krt5^+^ cells < 80% and ≥ 40%; (3) Pattern C, the ratio of p63^+^ cells to Krt5^+^ cells < 40%; and (4) Pattern D, both p63^+^ and Krt5^+^ cells are absent in nasal epithelium.

### Quantitative real-time polymerase chain reaction (qRT-PCR)

Total RNA was extracted from frozen IT tissues using the mirVana miRNA Isolation Kit (Life Technologies). Then, 1 μg of total RNA was reverse transcribed into cDNA using the Maxima Reverse Transcriptase Kit (Thermo Fisher Scientific) according to manufacturer’s protocol. Acet.α-tubulin, MUC5AC, p63, Krt5, and Ki67 mRNA levels were detected by qRT-PCR (SYBR Green, Promega, Madison, WI, USA). Relative gene expression was calculated using the comparative 2^–ΔΔCt^ method, normalized against the housekeeping gene glyceraldehyde 3-phosphate dehydrogenase (GAPDH). Acet.α-tubulin, MUC5AC, p63, Krt5, Ki67, and GAPDH amplification was performed with the following primers: Acet.α-tubulin forward (5′- CTTTGTATTTGGTCAGTCTGG-3′), Acet.α-tubulin reverse (5′- ATCTTGCTGATAAGGAGAGTG -3′); MUC5AC forward (5′- AATGGTGGAGATTT-TGACAC-3′), MUC5AC reverse (5′-TTCTTGTTCAGGCAAATCAG-3′); p63 forward (5′-CAGC- CTATATGTTCAGTTCAG-3′), p63 reverse (5′-CAGTCCATGCTAATCTCAATC-3′); Krt5 for-ward (5′- TGGAAGACTTCAAGAACAAG-3′), Krt5 reverse (5′-ATGTAGGCAGCATCTACATC-3′); Ki67 forward (5′-AATGGTGGAGATTT-TGACAC-3′), Ki67 reverse (5′-TTCTTGTTCAGG- CAAATCAG-3′).

### Statistical analysis

GraphPad Prism 7.0 (GraphPad Inc., USA) were employed for statistical analyses and figures. A Kruskal-Wallis test, followed by a Dunn’s multiple comparisons test were used to compare the expression levels of p63^+^Krt5^+^ and Ki67^+^ cells, and the ratio of The ratio of Ki67^+^ cells to p63^+^Krt5^+^ cells and p63^+^ cells to Krt5^+^ cells among the three groups (all variables were not normally distributed). A Mann-Whitney test was used to compare the expression levels of p63^+^Krt5^+^ and Ki67^+^ cells, and the ratio of Ki67^+^ cells to p63^+^Krt5^+^ cells and p63^+^ cells to Krt5^+^ cells between SM epithelium and non-SM epithelium in patients with CRSr. The Mann-Whitney 2-tailed nonparametric test was used to compare Acet.α-tubulin, MUC5AC, p63, Krt5, and Ki67 mRNA expression between control and CRSr, and between SM epithelium and non-SM epithelium in patients with CRSr, respectively.

## Results

### Clinical characteristics of subject

The clinical characteristics of controls, patients with CRSsNP and patients with CRSr are summarized in Table 1. The median time interval between RT and sample collection was 3 years (range of 1 to 17 years). Of the 36 NPC patients with CRSr, 2 cases (5.6%), 5 cases (13.9%), 16 cases (44.4%), and 13 cases (36.1%) were reclassified as stage I, II, III, and Iva, respectively according to the 7th edition AJCC/UICC staging system. All of the biopsies from controls showed healthy nasal epithelium. Epithelial hyperplasia was present in 21.2% of patients with CRSsNP and in 27.8% of patients with CRSr, and goblet cell hyperplasia was found in 9.1% of patients with CRSsNP and in 13.9% of patients with CRSr. The prevalence of epithelial hyperplasia and goblet cell hyperplasia both showed no significant difference between patients with CRSsNP and patients with CRSr (*P* = 0.585; *P* = 0.711). Nevertheless, The prevalence of SM was was significantly higher in patients with CRSr (36.1%) as compared to patients with CRSsNP (6.1%, *P* = 0.003).

### Cilia and goblet cell were both decreased in nasal epithelium of CRSr

**Fig. 1:**
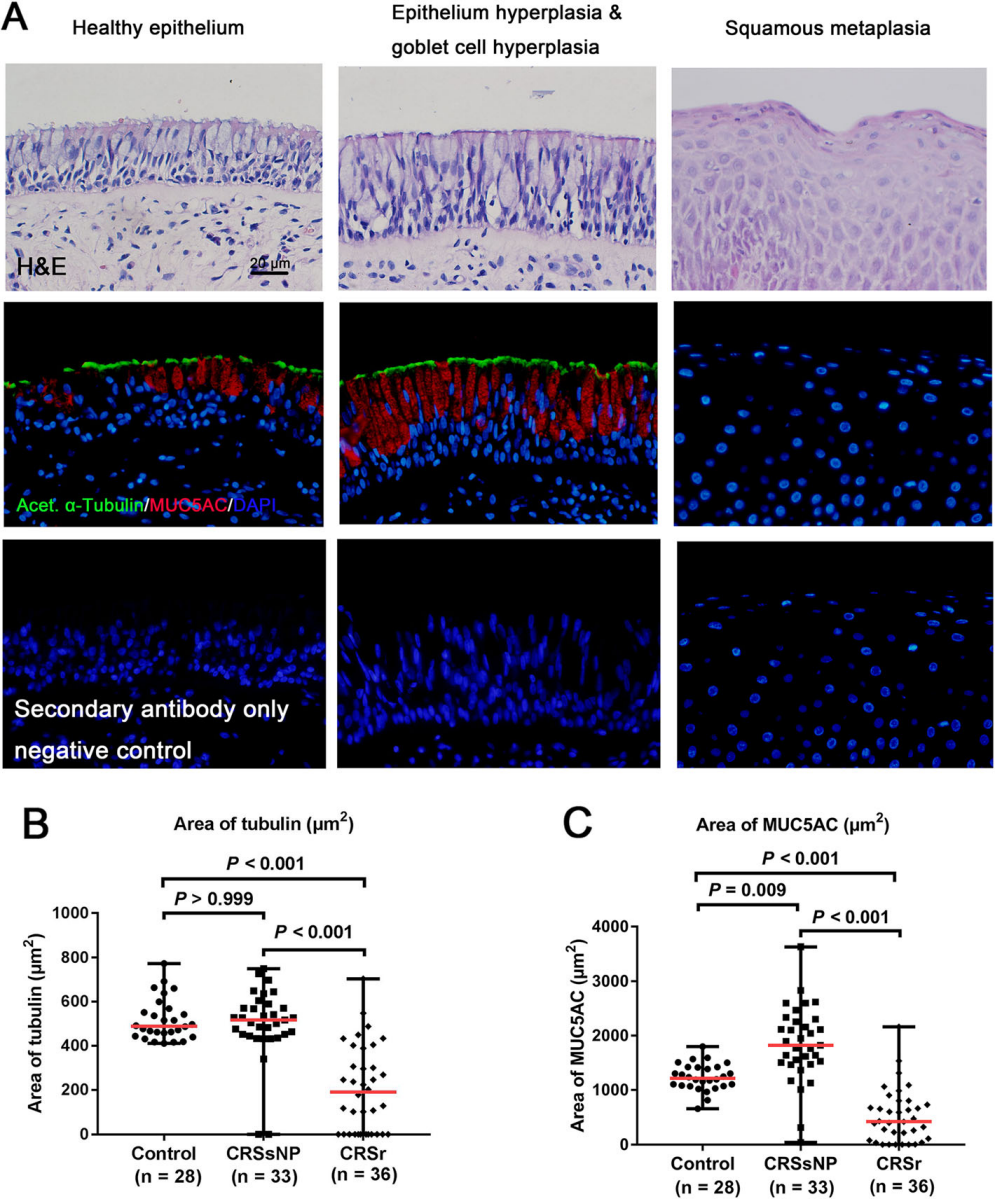
**a**. Double IF staining of acet. α-tubulin (green) and MUC5AC (red) in nasal epithelium (× 400 magnification; nucleus stained in blue; scale bar = 20 μm). Negative control staining was performed using secondary antibody only. **b**. The expression levels of acet. α-tubulin compared among the three groups. **c**. The expression levels of MUC5AC compared among the three groups

Double IF staining of acet. α-tubulin and MUC5AC was performed to assess cilia and goblet cell in nasal epithelium (Fig. 1a). The acet. α-tubulin area and MUC5AC area in nasal epithelium both showed significant difference among the three groups (both *P* < 0.001). The acet. α-tubulin area was decreased significantly in the nasal epithelium from patients with CRSr (190.1 μm^2^, 0–703.9 μm^2^) compared to both patients with CRSsNP (517.1 μm^2^, 0–749.5 μm^2^, *P* < 0.001) and controls (488.8 μm^2^, 411.6–772.8 μm^2^, *P < 0.001*), and showed no significant difference between patients with CRSsNP and controls (*P* > 0.999) (Fig. 1b). The MUC5AC area was decreased significantly in nasal epithelium from patients with CRSr (425.6 μm^2^, 0–2162.0 μm^2^, *P* < 0.001) and was increased significantly in nasal epithelium from patients with CRSsNP (1821.0 μm^2^, 40.58–3632.0 μm^2^, *P* < 0.001) compared to controls (1216.0 μm^2^, 655.9–1800.0 μm^2^) (Fig. 1c). Additionally, Acet. α-tubulin and MUC5AC mRNA expression were consistent with protein data where both were decreased significantly in IT tissue from patients with CRSr (*n* = 20) compared with that from control subjects (*n* = 6) (*P* = 0.030, *P* = 0.013) (Fig. S1A and B).

### p63^+^Krt5^+^ cells were increased in nasal epithelium of CRSr

**Fig. 2:**
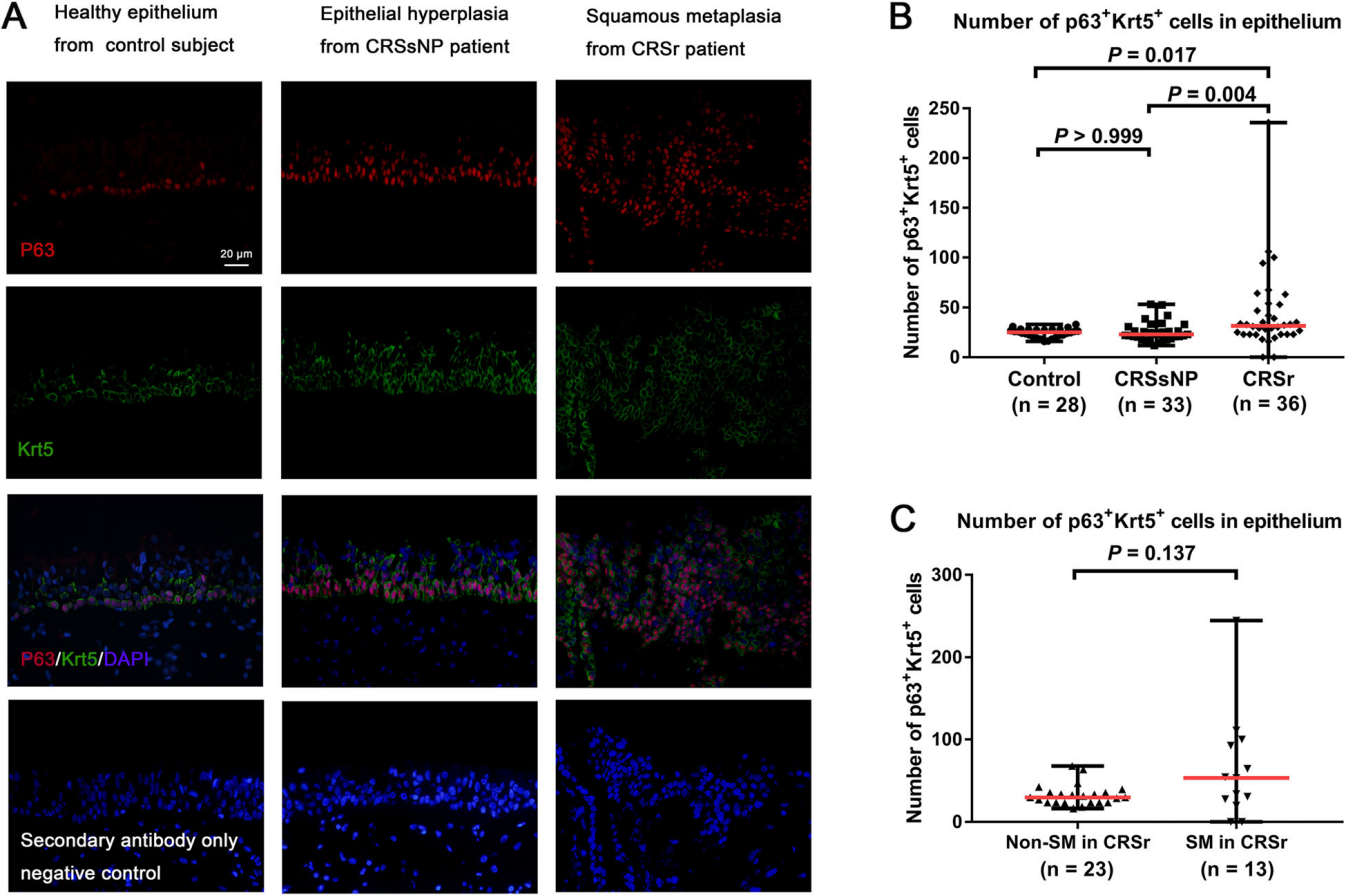
**a**. Double IF staining of p63 (red) and Krt5 (green) in nasal epithelium from controls, CRSsNP, CRSr. (× 400 magnification; nucleus stained in blue; scale bar = 20 μm). Negative control staining was performed using secondary antibody only. **b**. The expression levels of p63^+^Krt5^+^ cells compared among the three groups. **c**. The expression levels of p63^+^Krt5^+^ cells compared between SM cases and non-SM cases in CRSr group

Double IF staining of p63 and Krt5 was performed to assess the expression levels of p63^+^Krt5^+^ cells in nasal epithelium from all subjects (Fig. 2a). The total number of p63^+^Krt5^+^ cells in nasal epithelium showed significant difference among the three groups (*P* = 0.002). The total number of p63^+^Krt5^+^ cells was increased significantly in the nasal epithelium from patients with CRSr (31.67, 0–235.7) compared to both patients with CRSsNP (23.0, 11.67–53.33, *P* = 0.004) and controls (25.17, 16.0–33.0, *P* = 0.017), and showed no significant difference between patients with CRSsNP and controls (*P* > 0.999) (Fig. 2b). Interestingly, p63 mRNA expression was decreased significantly and Krt5 mRNA expression show no difference in IT tissue from patients with CRSr (*n* = 20) compared with that from control subjects (*n* = 6) (*P* = 0.013, *P* = 0.196) (Fig. S1C and E), potentially due to feedback against the increased positive cell numbers. In patients with CRSr, the total number of p63^+^Krt5^+^ cells were higher in SM epithelium (53.0, 0–244.30) compared to non-SM epithelium (29.33, 23.0–67.67, *P* = 0.137) (Fig. 2c); while p63 and Krt5 mRNA expression both showed no significant difference between non-SM cases (*n* = 14) and SM cases (n = 6) (*P* = 0.312, *P* = 0.494) (Fig. S1D and F).

### Ki67^+^ cells were increased in nasal epithelium of CRSr and CRSsNP

**Fig. 3:**
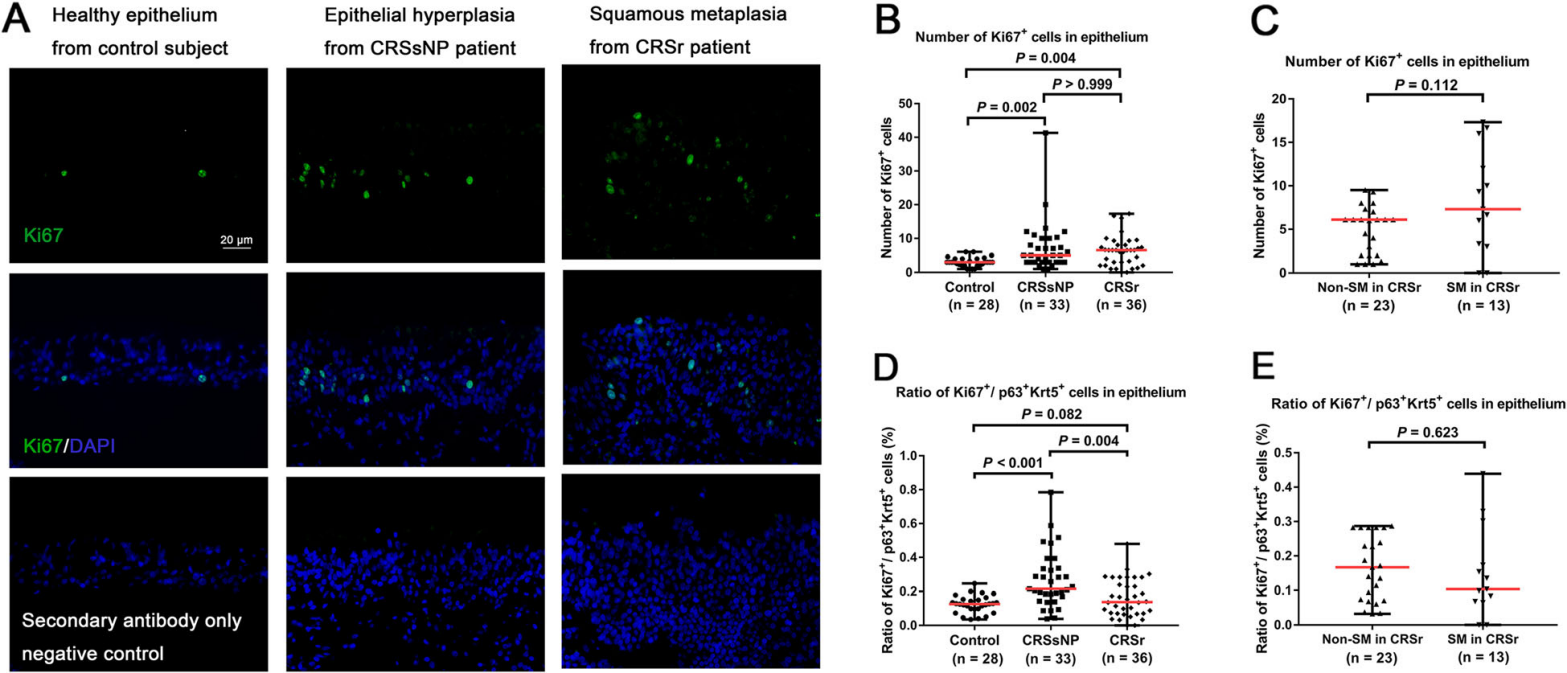
**a**. IF staining of Ki67 (green) in nasal epithelium from controls, CRSsNP, and CRSr. (× 400 magnification; nucleus stained in blue; scale bar = 20 μm). Negative control staining was performed using secondary antibody only. **b**. The expression levels of Ki67^+^ cells compared among the three groups. **c**. The expression levels of Ki67^+^ cells compared between SM cases and non-SM cases in CRSr group. **d**. The ratio of Ki67^+^ cells to p63^+^Krt5^+^ cells compared among the three groups. **e**. The ratio of Ki67^+^ cells to p63^+^Krt5^+^ cells compared between SM cases and non-SM cases in CRSr group

The total number of Ki67^+^ cells (Fig. 3a) in nasal epithelium showed significant difference among the three groups (*P* < 0.001). The total number of The Ki67^+^ cells was increased significantly in nasal epithelium from both patients with CRSr (6.50, 0–17.33, *P* = 0.002) and patients with CRSsNP (5.0, 1.0–41.33, *P* = 0.004) compared to controls (3.0, 1.0–6.0), but showed no significant difference between patients with CRSr and patients with CRSsNP (*P* > 0.999) (Fig. 3b). Conversely, Ki67 mRNA expression was decreased significantly in IT tissue from patients with CRSr (*n* = 20) compared with that from control subjects (*n* = 6) (*P* = 0.011) (Fig. S1G). In patients with CRSr, the total number of Ki67^+^ cells showed no significant difference between SM epithelium(7.30, 0–17.33) and non-SM epithelium (6.10, 1.0–9.50, *P* = 0.112) (Fig. 3c); Ki67 mRNA expression also showed no significant difference between non-SM cases (*n* = 14) and SM cases (*n* = 6) (*P* = 0.312) (Fig. S1H).

### The ratio of Ki67^+^ cells to p63^+^Krt5^+^ cells was decreased in nasal epithelium of CRSr

The ratio of Ki67^+^ cells to p63^+^Krt5^+^ cells was calculated to assess the proliferative capacity of basal cells in nasal epithelium, which showed significant difference among the three groups (*P* < 0.001). The ratio of Ki67^+^ cells to p63^+^Krt5^+^ cells was significantly higher in nasal epithelium from patients with CRSsNP(21.43%, 3.85–78.47%) compared to both patients with CRSr (13.78%, 0–48.0%, *P* = 0.004) and controls (12.61%, 3.53–24.66%, *P* < 0.001), and showed no significant difference between patients with CRSr and controls (*P* = 0.822) (Fig. 3d). In patients with CRSr, the ratio of Ki67^+^ cells to p63^+^Krt5^+^ cells was slightly lower in SM epithelium (10.35%, 0–43.91%) compared to non-SM epithelium(16.67%, 3.23–28.68%, *P* = 0.643) (Fig. 3e).

### The ratio of p63^+^ cells to Krt5^+^ cells was decreased in SM epithelium

**Fig. 4:**
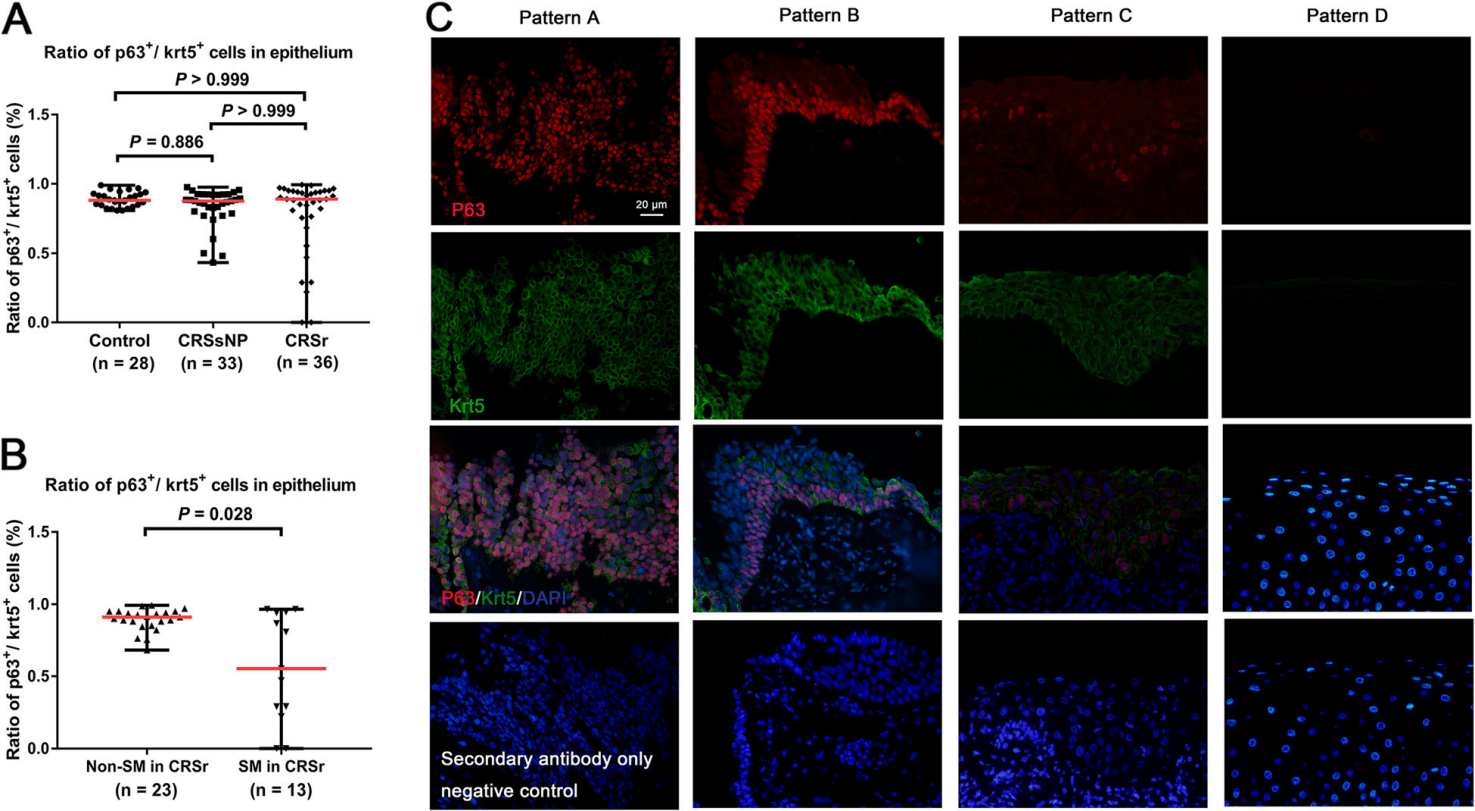
**a**. The ratio of p63^+^ cells to Krt5^+^ cells compared among the three groups. **b**. The ratio of p63^+^ cells to Krt5^+^ cells compared between SM cases and non-SM cases in CRSr group. **c**. SM patterns based on the ratio of p63^+^ cells to Krt5^+^ cells: (1) Pattern A, the ratio ≥ 80%; (2) Pattern B, the ratio < 80% and ≥ 40%; (3) Pattern C, the ratio < 40%; and (4) Pattern D, both p63^+^ and Krt5^+^ cells are absent in nasal epithelium. Negative control staining was performed using secondary antibody only

The ratio of p63^+^ cells to Krt5^+^ cells in nasal epithelium from controls(88.01%, 80.71–98.98%), patients with CRSsNP (87.50%, 43.30–97.56%), and patients with CRSr (88.89%, 0–99.35%) showed no significant difference (*P* = 0.548) (Fig. 4a). However, in patients with CRSr, the ratio of p63^+^ cells to Krt5^+^ cells were significantly lower in SM epithelium (55.35%, 0–96.57%) compared to non-SM epithelium (91.10%, 68.14–99.35%, *P* = 0.027) (Fig. 4b). A total of 13 cases in patients with CRSr represented SM in nasal epithelium. In these 13 cases, SM pattern A, B, C, and D (Fig. 4c) were found in 6 cases (46.1%), 2 cases (15.4%), 3 cases (23.1%), and 2 cases (15.4%) according to our classification system.

## Discussion

Extensive damage and secondary SM in nasal epithelium were common pathological features in patients with CRSr. Epithelial sloughing, ciliary loss, intercellular and intracellular vacuolization, and ciliary dysmorphism were present in histologic examination of nasal epithelium after RT. Greater SM was also observed in histologic examination of nasal biopsied tissue from patients with CRSr compared to CRS patients without RT [19]. In this study, we demonstrated for the first time that increased SM and expression of p63^+^Krt5^+^ basal cells, together with loss of cilia and goblet cells, in nasal epithelium from patients with CRSr compared to both patients with CRSsNP and healthy controls. Furthermore, in patients with CRSr, increased expression of p63^+^Krt5^+^ basal cells were present in SM epithelium rather than non-SM epithelium. Together these data suggest that aberrant proliferation of basal cells may contribute to SM in nasal epithelium from patients with CRSr. The results of this study provide new insights into the molecular mechanism underlying epithelial remodeling in CRSr, which may be helpful to develop novel candidate targets for preventing SM process and restoring epithelial barrier function.

Squamous differentiation is an aberrant biological process in a number of tissues (e.g., trachea, bronchus, uterus, and bladder), and toxic and mechanical injury have been reported to induce SM in these tissues [20]. Previous studies have identified that smoking is an independent risk factor for SM in nasal polyps and Chronic obstructive pulmonary disease, and oxidative stress induce by cigarette smoke had been postulated as cause [12, 21]. Radiotherapy is cytotoxic to rapidly multiplying cancer cells but also affects proliferating normal cells in the mucosa. Radiation-induced mucositis is initiated by direct injury to epithelial cells and the underlying submucosal tissue [22]. Histologic examination of post radiation nasal tissue has shown increased fibrosis in the lamina propria, epithelial sloughing, ciliary loss, and intercellular and intracellular vacuolization, resulting in destruction of nasal epithelial barrier and subsequent epithelial remodeling [19]. In the present study, SM occurred more frequently in nasal epithelium from CRSr patients compared to CRSsNP patients without RT. Therefore, radiotherapy may induce direct damage to epithelial cells and increase the likelihood of subsequent SM process in the nasal epithelium from patients with CRSr.

Basal cells in airway epithelium are considered to have stem/progenitor properties, which can self-renew and differentiate into other nasal epithelial cell types, such as goblet cells and columnar ciliated and non-ciliated cells [23]. In normal airway epithelium, p63 proteins are situated in the nuclei of basal cells, Krt5 protein are presented in the cytoskeleton of basal cells, and p63^+^Krt5^+^ basal cells are usually regarded as the stem cells [24–27]. The balance between the rate of proliferation and differentiation of basal cells is necessary to maintain the normal structure of the epithelium [28]. However, basal cells appear to undergo hyperproliferation and show increased squamous differentiation in chronic inflammatory situations [29]. In this study, hyperproliferation of p63^+^Krt5^+^ basal cells were observed in nasal epithelium (especially in SM areas) from patients with CRSr but not in nasal epithelium from patients with CRSsNP. The results indicate that aberrant proliferation of p63^+^Krt5^+^ basal cells is an important histopathologic characteristic of squamous metaplastic epithelium from patients with CRSr, and the mechanism of nasal epithelial remodeling in response to radiation damage may be different from that only caused by infection and inflammation. Instead, toxic injury from radiation was postulated to be more similar to damage arising from inhalant exposures such as cigarette smoking, where both initiated a multistage process of metaplastic transformation in the epithelium [19]. Hence, radiation damage may similarly promotes basal cells within the pseudostratified epithelium to re-enter the cell cycle, resulting in hyperproliferative cells that drive the increase of basal cells in squamous metaplastic epithelium [21]. Shaykhiev et al. further showed that the transition of the epithelium towards SM may be via the activation of the epidermal growth factor (EGF) / EGF receptor (EGFR) pathway due to oxidative stress induce by cigarette smoke damage [30], which may also be applied to radiation damage.

Ki-67 is a nuclear protein present during all active phases of the cell cycle (G1, S, G2, and M), but is absent in resting cells (G0) [31]. Ki67^+^ cells are mainly located along the basal layer of nasal epithelium and specifically located in basal cells, indicating that cell proliferation in nasal epithelium mainly occurred in basal cells [16]. Ki67^+^ cells and S-phase cells were both increased significantly in epithelium from NPs, suggesting cell proliferation is increased in epithelium from NPs caused by inflammatory mediators via repair processes of epithelial damage [32, 33]. In this study, Ki67^+^ cells were both increased significantly in nasal epithelium from patients with CRSr and CRSsNP compared to controls, but it showed no significant difference between patients with CRSr and CRSsNP. Furthermore, the ratio of Ki67^+^ cells to p63^+^Krt5^+^ cells were significantly lower in nasal epithelium from patients with CRSr compared to patients with CRSsNP, indicating that more proliferating basal cells in nasal epithelium after radiation have lost their regenerative property. The proliferating basal cells tend to differentiate into squamous metaplastic cells but not normal nasal epithelial cells after radiation damage. Hence, it is likely that the absence of normal epithelium resulted in the constant activation of repair signals, which in turn further aggravate hyperproliferation of basal cells [20, 28]. Interestingly, suppressed mRNA levels of p63 and Ki67 further suggest inherent abnormality basal and proliferative gene expression in CRSr tissues that may further contribute to the SM pathogenesis. It may also indicate a feedback loop to reverse the process, but may have been rendered ineffective due to other post translational modifications which results in the retention of damaged and abnormal basal cells. In addition, the aberrant levels of basal cell and proliferative markers then in turn affected the expression of Acet.α-tubulin and MUC5AC, which then resulted in the reduction of normal epithelial differentiation, contributing to the epithelial damage and absence of fully differentiated epithelium. This vicious cycle of radiation damage and aberrant repair process may result in basal cells depletion and full formation of SM in nasal epithelium, which warrant future investigation to elucidate the actual mechanisms that resulted in the aberrant levels of gene and protein expression that may contribute to the pathology and SM process of CRSr.

Furthermore, our study found that p63^+^ cells number was slightly more than Krt5^+^ cells number in healthy nasal epithelium, and the ratio of p63^+^ cells to Krt5^+^ cells was generally more than 80%. Interestingly, the ratio of p63^+^ cells to Krt5^+^ cells tended to significantly decline in the SM process, and p63^+^ cells and Krt5^+^ cells both depleted in fully SM epithelium. This phenomenon suggested an asynchronous degeneration of basal cells structures in the SM process, which developed from the inside(nuclei) to the outside(cytoskeleton). Different SM patterns based on the ratio of p63^+^ cells to Krt5^+^ cells may represent different stages of SM process in nasal epithelium, but the intrinsic mechanism remains unclear and further studies are needed to verify this relationship.

There are still some limitations of this study. Firstly, the mRNA expression levels of p63, Krt5 and Ki67 between the three groups were obtained from a fraction of the subjects and were compared only between controls and CRSr patients. Nevertheless, the mRNA levels comparison does indicate potential CRSr SM process mechanism at the post translational level. Secondly, we did not record the total symptom score or the visual analogue scale of chronic rhinosinusitis, which would be useful for assessing symptom severity in patients with CRSsNP and CRSr. Lastly, we did not manage to eliminate the effect of chemotherapy on epithelial damage in the patients with CRSr recruited in this study, because most of these patients underwent concurrent chemotherapy during RT.

The results of this study showed that aberrant proliferation and differentiation of stem/progenitor cells were implicated in the SM process in nasal epithelium after RT, and early detection of these morphological changes is important to facilitate early intervention to better manage the pathology. In addition, it is also important to ascertain in the future if the dosage of radiation is directly correlated to degree of epithelial damage in order to improve treatment prognosis. Recently, reconstructed tissue with basal cells has been applied to treat a patient with airway stenosis [34]. Whether stem/progenitor cells regeneration therapy is an effective way to restore the structure and function of nasal epithelium from patients with CRSr still requires more experimental evidence. In further studies, it is important to explore the molecular mechanisms, pathways and dose-dependent effect of the RT in contributing to SM in patients with CRSr, which will serve as a foundation for the development of stem/progenitor cells mediated epithelial regeneration therapy in CRSr patients.

## Conclusions

In conclusion, SM is increased in the nasal epithelium from patients with CRSr, potentially due to aberrant proliferation of p63^+^Krt5^+^ basal cells are important histopathologic features in squamous metaplastic epithelium after radiation.

## Supplementary information

**Additional file 1: Figure S1.** A. The mRNA level of acet. α-tubulin between controls and patients with CRSr. B. The mRNA level of MUC5AC between controls and patients with CRSr. C. The mRNA level of p63 between controls and patients with CRSr. D. The mRNA level of p63 between SM cases and non-SM cases in CRSr group. E. The mRNA level of Krt5 between controls and patients with CRSr. F. The mRNA level of Krt5 between SM cases and non-SM cases in CRSr group. G. The mRNA level of Ki67 between controls and patients with CRSr. H. The mRNA level of Ki67 between SM cases and non-SM cases in CRSr group.

## Data Availability

All data generated or analyzed during this study are included in this published article.

## Abbreviations

NPC: Nasopharyngeal carcinoma
RT: radiotherapy
CRS: Chronic rhinosinusitis
CRSr: radiation-induced chronic rhinosinusitis
SM: Squamous metaplasia
NPs: nasal polyps
IF: Immunofluoresence
CRSsNP: CRS without nasal polyps
IMRT: intensity-modulated radiotherapy
IT: Inferior turbinate
