# Supplementary figures and images for: p63+Krt5+ basal cells are increased in the squamous metaplastic epithelium of patients with radiation-induced chronic Rhinosinusitis
Source: Radiat Oncol. 2020 Sep 25;15:222. doi: 10.1186/s13014-020-01656-7 (PMC7517817; doi:10.1186/s13014-020-01656-7)

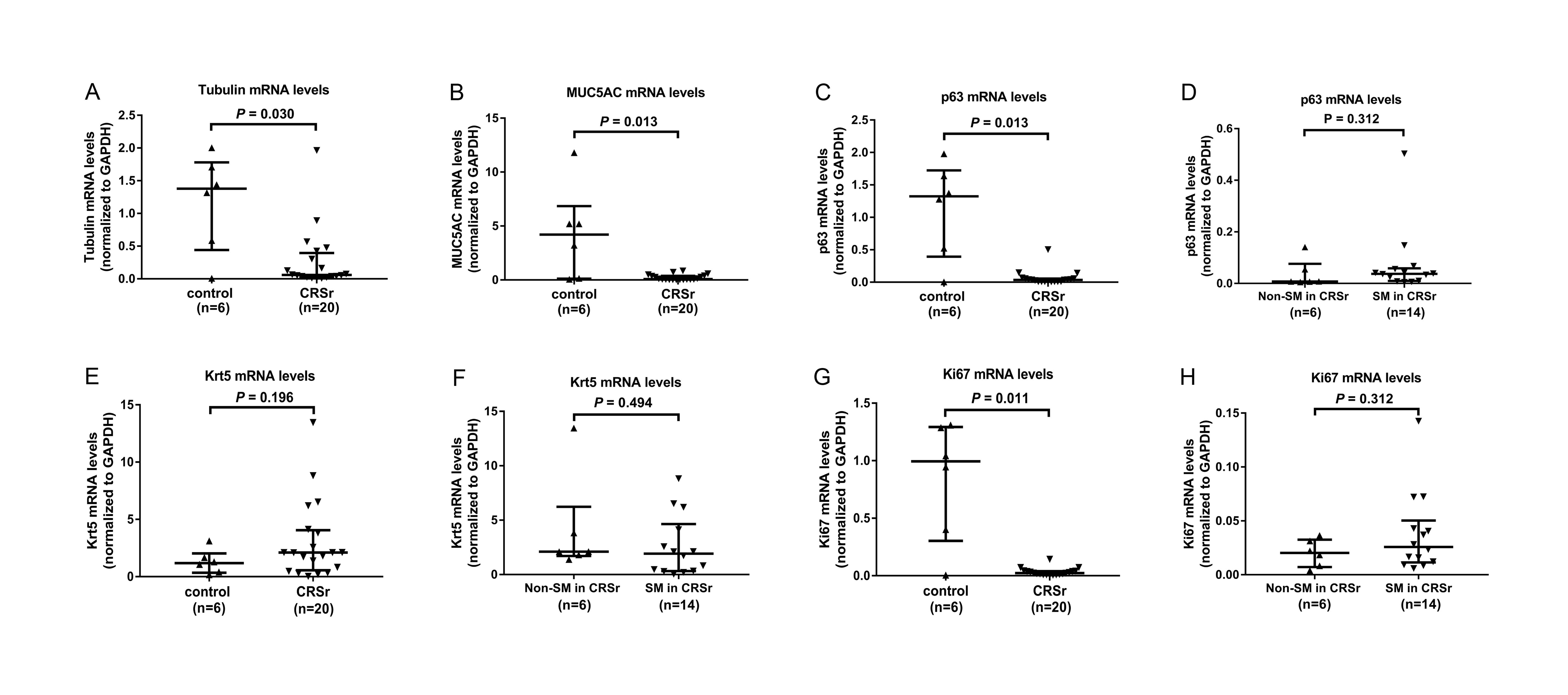

Supplement: Supplementary file 1 — Additional file 1: Figure S1. A. The mRNA level of acet. α-tubulin between controls and patients with CRSr. B. The mRNA level of MUC5AC between controls and patients with CRSr. C. The mRNA level of p63 between controls and patients with CRSr. D. The mRNA level of p63 between SM cases and non-SM cases in CRSr group. E. The mRNA level of Krt5 between controls and patients with CRSr. F. The mRNA level of Krt5 between SM cases and non-SM cases in CRSr group. G. The mRNA level of Ki67 between controls and patients with CRSr. H. The mRNA level of Ki67 between SM cases and non-SM cases in CRSr group. [file 13014_2020_1656_MOESM1_ESM.tif]
